# Radioprotective Potential of a Polyphenol-Rich Extract Blend: Preclinical Evaluation in Female Balb/c Mice Exposed to Ionizing Radiation

**DOI:** 10.3390/ijms26209972

**Published:** 2025-10-14

**Authors:** Karolina Niska, Patrycja Bloch, Paulina Karolina Kowalczyk, Katarzyna Zima, Michalina Gramatyka, Tomasz Cichoń, Michał Dobkowski, Krzysztof Lemke, Barbara Khaidakov

**Affiliations:** 1 R&D Department, AronPharma Ltd., Trzy Lipy Street 3, 80-172 Gdansk, Poland; patrycja.bloch@aronpharma.pl (P.B.); michal.dobkowski@aronpharma.pl (M.D.); krzysztof.lemke@aronpharma.pl (K.L.); 2 3P-Medicine Laboratory, Medical University of Gdansk, Dębinki 7, 80-211 Gdansk, Poland; kowalczyk.paulina@gumed.edu.pl; 3 Department of Physiology, Medical University of Gdansk, Dębinki 1, 80-211 Gdansk, Poland; katarzyna.zima@gumed.edu.pl; 4 Maria Skłodowska-Curie National Research Institute of Oncology, Gliwice Branch, Wybrzeże Armii Krajowej Street 15, 44-101 Gliwice, Poland; michalina.gramatyka@gliwice.nio.gov.pl (M.G.); tomasz.cichon@gliwice.nio.gov.pl (T.C.)

**Keywords:** polyphenols, ionizing radiation, acute radiation, radioprotection, oxidative stress, immune modulation, inflammation, DNA damage

## Abstract

Radiation is widely used in cancer therapy but also damages healthy tissues through oxidative stress or inflammation. In addition to cancer patients, many professionals are occupationally exposed to ionizing radiation (IR). Natural compounds, particularly polyphenols, have been increasingly investigated as potential radioprotective agents to minimize side effects in both patients and occupationally exposed individuals. This study evaluated the radioprotective effects of a polyphenol-rich extract blend derived from chokeberry, elderberry, blackcurrant, and evening primrose in female Balb/c mice exposed to acute IR. The animals were pre-treated with the blend (100 mg/kg) for 7 days prior to whole-body IR at 6 Gy. Hematological parameters, immune cell viability, TNF-α level, gene expression, lipid peroxidation, and tissue morphology were assessed by hematology analysis, flow cytometry, ELISA, qRT-PCR, MDA assay, and histology. IR significantly reduced leukocyte (3.22-fold; *p* < 0.0001) and platelet counts (1.37-fold; *p* < 0.0001), increased TNF-α levels (53.93%; *p* < 0.0001), and elevated oxidative stress. Pre-treatment with the blend restored hematological parameters, reduced pro-inflammatory cytokines, and normalized genes regulating oxidative stress and apoptosis. Histology confirmed preserved liver and kidney structures compared with irradiated controls. These findings highlight the polyphenol-rich extract blend as a promising natural radioprotective agent by modulating immune responses, reducing oxidative stress, and preserving tissue integrity.

## 1. Introduction

Radiotherapy, alongside surgery and chemotherapy, constitutes a cornerstone of comprehensive cancer care. Cancer remains the leading cause of death worldwide, with the highest mortality rates observed in low- and middle-income countries. According to the American Cancer Society’s Global Cancer Statistics 2024 report, approximately 20 million new cancer cases were diagnosed in 2022, resulting in around 9.7 million deaths. Furthermore, the global cancer burden is expected to increase substantially, reaching an estimated 35 million new cases by 2050, a 77% rise compared to 2022 [1]. It is estimated that 30 to 50% of all cancer patients receive radiotherapy, either as standalone treatment or in combination with other therapies [2].

During radiotherapy, exposure to ionizing radiation (IR) directly and indirectly (by increasing the level of reactive oxygen species (ROS)) damages cellular structures and components such as lipids, proteins, and DNA, ultimately leading to inhibition of cell proliferation and death [3,4]. Exposure to IR involves both therapeutic benefits and potential risks to healthy tissues. Although cancer cells are more susceptible to radiation-induced damage due to their rapid proliferation and limited DNA repair mechanisms, healthy tissues may also be affected, resulting in potential side effects [5]. The severity of those side effects depends on many factors including radiation dose, duration and intensity of exposure, exposed area, biological sensitivity of various tissues, and even individual genetic background [6]. Acute damage predominantly affects rapidly dividing cells, such as those of bone marrow and lymphoid organs [7], whereas chronic IR exposure (usually associated with long-term and low-dose exposure) can induce fibrosis in metabolically active organs like the liver and kidneys. This fibrotic process is driven by oxidative stress, cytokine dysregulation, and aberrant intracellular signaling, leading to myofibroblast activation and extracellular matrix accumulation [8].

IR plays a crucial role in medicine, not only in cancer radiotherapy but also in diagnostics, biomedical research, and various industrial applications. With an increased risk of IR exposure, a focus on radiation safety is crucial not only for patients and healthcare professionals but also for specialists in other fields where IR is used [6]. According to the United Nations Scientific Committee on the Effects of Atomic Radiation, millions of workers worldwide are occupationally exposed to IR [9]. Epidemiological studies identified a strong correlation between such exposure and the development of chronic conditions, including cancer and cardiovascular diseases [10].

Given the widespread use of IR, developing effective radioprotective strategies is essential to mitigate both acute and long-term health effects. While synthetic radioprotectors like amifostine have demonstrated protective effects in cancer patients, their clinical use is often limited by side effects such as nausea, hypotension, and organ toxicity [11]. This highlights the need for safer alternatives for the protection of healthy tissues. Among various agents, polyphenolic compounds, such as flavonoids (e.g., quercetin, catechins, genistein or silibinin), anthocyanins, stilbenes (e.g., resveratrol), gallic acid, and curcumin, which are naturally occurring plant-derived antioxidants, have shown promising radioprotective potential. These agents exert multifaceted effects, including scavenging reactive oxygen and nitrogen species, suppressing pro-inflammatory signaling pathways (such as NF-κB activation), preserving mitochondrial function, and supporting DNA repair mechanisms [12,13,14]. Additionally, polyphenols were proved to protect against radiation through stimulation of hematopoietic recovery and modulating immune responses [13]. Numerous in vivo studies have confirmed the radioprotective properties of polyphenolic compounds such as quercetin, curcumin, caffeic acid, and resveratrol against radiation-induced toxicities [12,13,15,16,17]. Anthocyanins from *Lonicera caerulea* var. *edulis* protected mice against gamma radiation by scavenging free radicals and enhancing antioxidant enzyme activity, partially restoring spleen and thymus function [18]. Importantly, compared to synthetic compounds, natural polyphenols generally display a favorable safety profile, making them suitable candidates for long-term use or dietary supplementation. Among natural sources, berries such as chokeberry (*Aronia melanocarpa* L.) [19], elderberry (*Sambucus nigra* L.) [20], and blackcurrant (*Ribes nigrum* L.) [21] are particularly rich in polyphenolic compounds, including anthocyanins, proanthocyanidins (PACs), flavonols, phenolic acids, and hydroxycinnamic acids. Evening primrose (*Oenothera biennis* L.), although primarily recognized as a source of omega fatty acids, also contains stable polyphenolic compounds, such as ellagic acid, gallic acid, and catechin [22]. The high levels of bioactive compounds in these plants confer a broad range of biological activities, including antioxidant, anti-inflammatory, cytoprotective, and immunomodulatory effects, as demonstrated in both in vitro and in vivo studies [20,23,24,25], indicating their potential as natural radioprotective agents. Notably, chokeberry extracts have been shown to protect the gastrointestinal tract from IR-induced damage [26]. Similarly, blackcurrant has been reported to exert genome-protective effects in vitro, reducing γ-irradiation-induced micronuclei formation, gene mutations, and oxidative stress [27]. However, the effectiveness of specific polyphenolic combinations in protecting against radiation-induced damage remains insufficiently explored.

The aim of this study was to evaluate the effects of a novel polyphenol-rich extract blend on biological responses following exposure to IR in female BALB/c mice. Through these investigations, we have further advanced our understanding of the protective role of polyphenols in modulating radiation-induced cellular damage. While this study primarily focuses on the acute effects of IR, it is also essential to investigate the long-term effects, which represent a significant direction for future research.

## 2. Results

Since no adverse effects or significant changes were observed in any of the performed assays in group II, which was supplemented only with the tested blend, the results for this group were compared only with the control group to demonstrate the lack of any effect. The description of the results focused primarily on comparing the impact of the blend on protection against IR, emphasizing the effects observed in the irradiated groups with or without supplementation. All results were normalized to the values obtained in the IR control group (set as 100% injury) and then expressed as a percentage change relative to IR to demonstrate the cytoprotective effect.

### 2.1. The Phytochemical Composition of Individual Extracts and Their Blend

Chromatographic analysis of the individual extracts demonstrated that chokeberry contains the following anthocyanins: cyanidin 3-O-galactoside (C-3-Gal) (64.68%), cyanidin 3-O-glucoside (C-3-Glu) (3.20%), cyanidin 3-O-arabinoside (C-3-Ara) (28.08%), cyanidin 3-xyloside (C-3-Xyl) (4.04%). In elderberry the predominant anthocyanins identified were cyanidin 3-sambubioside-5-O-glucoside (C-3-Sam-5-Glu) (9.41%), cyanidin-3,5-O-diglucoside (C-3,5-di-Glu) (7.88%), cyanidin 3-sambubioside (C-3-Sam) (43.19%), cyanidin 3-O-glucoside (C-3-Glu) (39.52%). In blackcurrant, the main anthocyanins detected were delphinidin-3-O-glucoside (D-3-Glu) (31.25%), delphinidin-3-rutinoside (D-3-Rut) (26.65%), cyanidin 3-O-glucoside (C-3-Glu) (20.38%), cyanidin 3-rutinoside (C-3-Rut) (21.72%).

**Figure 1:**
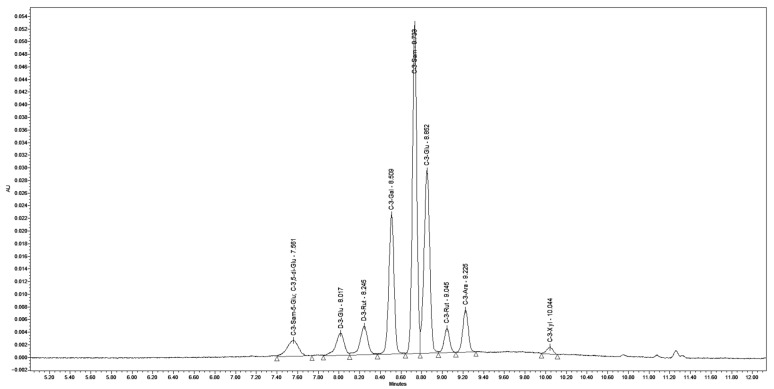
Chromatographic profile of the extract blend with identified anthocyanin compounds.

Chromatographic analysis of the blend confirmed the presence of the same anthocyanin compounds as those identified in the individual extracts (Figure 1). UV-Vis spectrophotometric analysis indicated a total anthocyanin content of 20.4% (*m*/*m*), which is detailed along with the individual anthocyanins and their respective contents in Table 1.

According to the supplier (Greenvit Ltd., Zambrów, Poland), the standardized polyphenol content in each extract was as follows: chokeberry—70.86%, evening primrose seeds—50.99%, elderberry—46.58%, and blackcurrant—47.11%. In the blend, the total phenolic content (TPC), determined using the Folin–Ciocalteu assay with caffeic acid as the calibration standard, was 54.74% (*m*/*m*) (Table 2).

### 2.2. Blood Analysis

**Figure 2:**
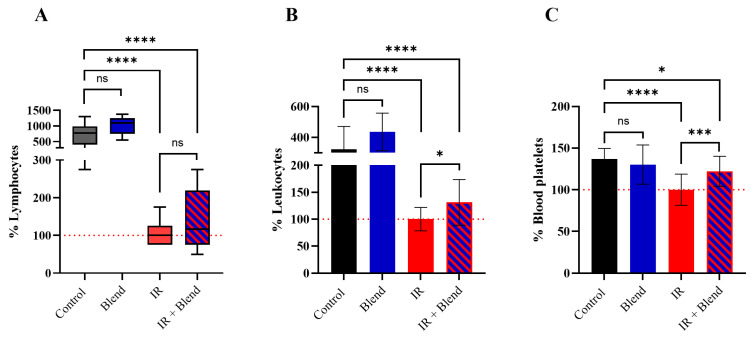
The morphological changes in blood collected from mice 24 h after exposure to IR: (**A**) lymphocyte, (**B**) leukocyte, and (**C**) blood platelet levels. The results are shown as a percentage of the values obtained in the IR control group (set as 100%) (*n* = 10). For graph (**A**), data are presented as box plot due to a non-normal distribution, and statistical comparisons were performed using the Mann–Whitney test. For graphs (**B**,**C**), data are expressed as the mean ± SD, and statistical analysis was conducted using Student’s *t*-test. Significance levels: **** *p* < 0.0001; *** *p* < 0.001; * *p* < 0.05; ns—not significant.

To investigate the impact of IR on blood morphology, blood samples were analyzed 24 h after mouse irradiation (Figure 2). Exposure to a single 6 Gy dose resulted in a significant decrease (*p* < 0.0001) in lymphocyte (Figure 2A), leukocyte (Figure 2B), and platelet (Figure 2C) levels compared to non-irradiated controls. In the group supplemented with the extract blend and exposed to IR, the observed decrease in white blood cells and platelets was mitigated compared to the IR-only group. Specifically, in the IR + Blend group, we observed higher levels of lymphocytes (38.36%; *p* = 0.2560), leukocytes (31.06%; *p* = 0.0177), and platelets (22.25%; *p* = 0.0006) compared to the IR-only group.

### 2.3. Flow Cytometry Analysis

**Figure 3:**
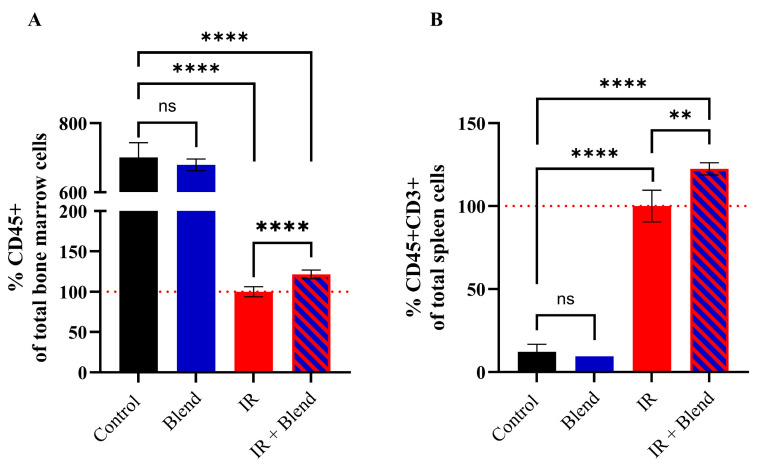
Flow cytometry-based detection of (**A**) CD45^+^ in bone marrow cells and (**B**) CD45^+^CD3^+^ in the spleen cells. The results are shown as a percentage of the values obtained in the IR control group (set as 100%) and presented as means ± SD (*n* = 6). Statistical analysis was conducted using Student’s *t*-test. Significance levels: **** *p* < 0.0001; ** *p* < 0.01; ns—not significant.

We performed flow cytometry analysis on mouse bone marrow and spleen cells to investigate how the irradiation affects immune cell populations and hematopoietic activity in extract blend supplemented and non-supplemented groups. The results showed a significant decrease in the level of CD45^+^ leukocytes in bone marrow cells after irradiation (Figure 3A). However, in the group supplemented with the extract blend and exposed to irradiation the decrease was smaller, and the level of CD45^+^ leukocytes was higher when compared to the IR-only group (21.39%; *p* < 0.0001). In the spleen, a significant increase in levels of T lymphocytes (CD45^+^CD3^+^ cells) was observed in both irradiated groups (*p* < 0.0001), with even higher level in supplemented group when compared to the IR-only group (22.4%; *p* = 0.0021) (Figure 3B).

### 2.4. TNF-α Measurement in Bone Marrow Cells

**Figure 4:**
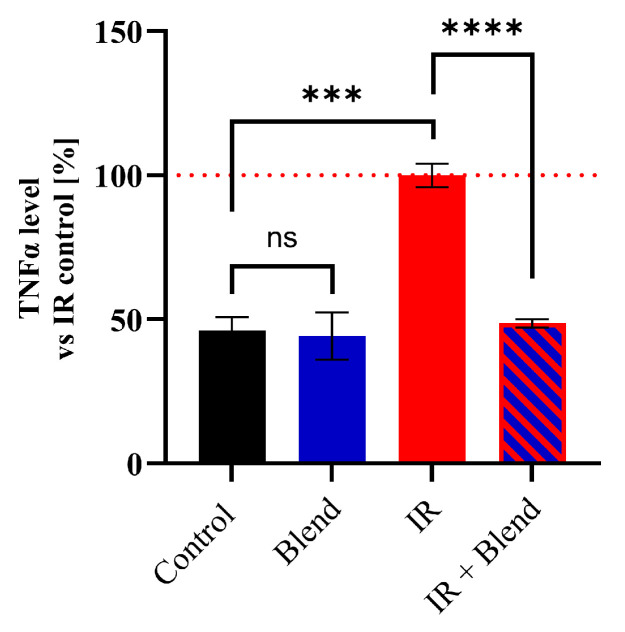
The level of TNF-α determined using the ELISA method in supernatants from bone marrow cell cultures stimulated with PMA and ionomycin for 5 h. The results are shown as a percentage of the values obtained in the IR control group (set as 100%) and presented as means ± SD of pooled mice from three independent replicates (*n* = 3). Statistical analysis was conducted using Student’s *t*-test. Significance levels: **** *p* < 0.0001; *** *p* < 0.001; ns—not significant.

To determine how IR and supplementation with the extract blend could affect the ability of BM cells to produce TNF-α following stimulation with PMA and ionomycin, TNF-α levels were measured in the supernatants of BM cells using ELISA (Figure 4). TNF-α levels significantly increased by 53.93% (*p* < 0.001) in the IR group compared to the negative control group. In contrast, in the group irradiated after supplementation with the extract blend, no increase in TNF-α level was observed.

### 2.5. Gene Expression Analysis

**Figure 5:**
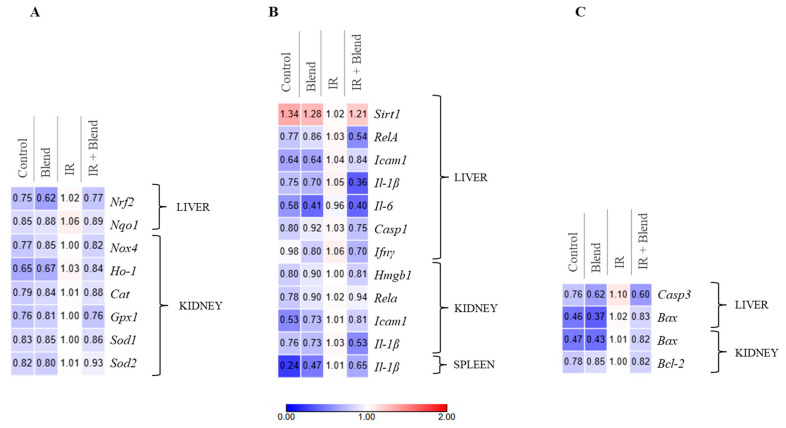
Gene expression associated with (**A**) oxidative stress, (**B**) inflammation, and (**C**) cell death. Gene expression levels were normalized to the reference gene, and relative quantification was performed using the 2^−ΔΔCt^ method. Blue color indicates decreased expression relative to the IR group, while red indicates increased expression relative to the IR group (*n* = 9).

Gene expression related to oxidative stress, inflammation, and cell death was analyzed in the liver, kidneys, and spleen isolated from mice. In the group exposed to IR, an upregulation of genes expression associated with oxidative stress was observed in the liver and kidneys (Figure 5A). In the liver, radiation triggered the activation of *Nrf2* (Nuclear factor erythroid 2-related factor 2) and the induction of the ROS-neutralizing enzyme *Nqo1* (NAD(P)H-quinone dehydrogenase 1). In the kidneys, oxidative stress induction was indicated by increased expression of the *Nox4* gene (NADPH oxidase 4) and a decreased expression of genes encoding the antioxidant enzymes, including *Ho-1* (Heme oxygenase), *Cat* (Catalase), *Gpx1* (Glutathione peroxidase 1), *Sod1* (superoxide dismutase 1), and *Sod2* (superoxide dismutase 2). In contrast, supplementation with the blend prevented these IR-induced alterations, maintaining the expression of oxidative stress-related genes at levels comparable to the non-irradiated control group, indicating a protective effect.

Additionally, in the IR group, we noted an increase in the expression of genes encoding inflammatory proteins in the liver, kidneys, and spleen (Figure 5B). Elevated expression of *Nf-κB* (the p65 subunit of the nuclear factor kappa B), *Icam-1* (Intercellular adhesion molecule 1), *Il-1β* (Interleukin 1β), *Il-6* (Interleukin 6) was detected in the liver and kidneys. Moreover, in the liver, we observed upregulation of *Casp-1* (caspase 1), and *Ifnγ* (Interferon gamma) and downregulation of the *Sirt1* gene, which encodes a negative regulator of immune response. In the kidneys, upregulation of *Hmgb-1* (High mobility group protein 1) expression was indicated, whereas in the spleen, only upregulation of *Il-1β* was detected. In contrast, compared to the IR-only group, in the experimental group irradiated after supplementation with the blend lower expression of pro-inflammatory genes was detected, along with an increased expression of *Sirt1* in the liver.

The analysis of genes associated with cell death showed that IR induced apoptosis (Figure 5C). In the liver, we observed increased expression of *Bax* (Bcl-2 Associated X-protein) and *Casp3* (Caspase 3), whereas in the kidneys, we observed upregulation of both *Bax* and *Bcl-2*. In the group that received supplementation before IR, the expression levels of these genes were lower compared to the irradiated group, indicating that supplementation helped restore the balance between pro- and anti-apoptotic pathways.

These findings suggest that supplementation with the extract blend may effectively prevent the upregulation of expression of genes associated with oxidative stress and cell death while mitigating inflammation by modulating immune response-related gene expression in both the liver and kidneys.

### 2.6. Assessment of Lipid Peroxidation

**Figure 6:**
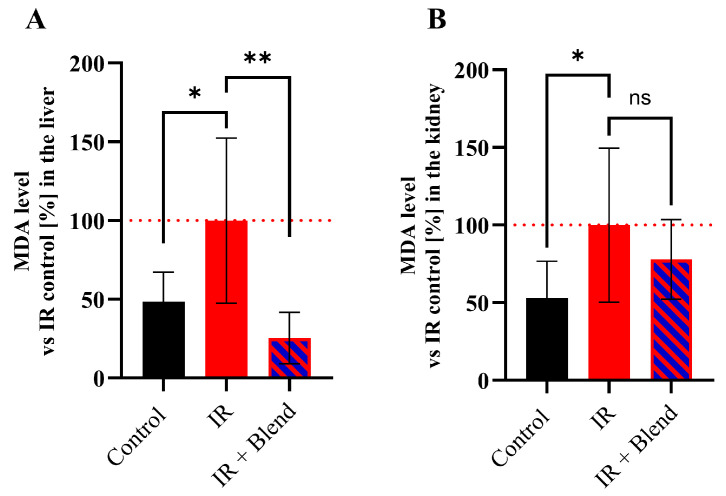
MDA concentration levels in mouse (**A**) liver and (**B**) kidneys. The results are presented as means ± SD (n = 9). The results are shown as a percentage of the values obtained in the IR control group (set as 100%) and presented as means ± SD (*n* = 9). Statistical analysis was conducted using Student’s *t*-test. Significance levels: ** *p* < 0.01; * *p* < 0.05; ns—not significant.

To assess oxidative stress, an analysis of lipid peroxidation through the measurement of MDA concentration in the liver and kidneys was conducted. The obtained results showed that IR induced the initiation of lipid peroxidation, as indicated by a significant increase in MDA levels in the IR group in both examined tissues—an increase of 51.66% in the liver (*p* = 0.0326) and 47.14% in the kidneys (*p* = 0.0395) compared to the negative control (Figure 6). Compared to the IR-only group, the supplemented group exhibited a 74.62% reduction in hepatic MDA levels (*p* = 0.0042), whereas the 22.13% decrease in renal MDA did not reach statistical significance (*p* = 0.3083).

### 2.7. Histological Evaluation

**Figure 7:**
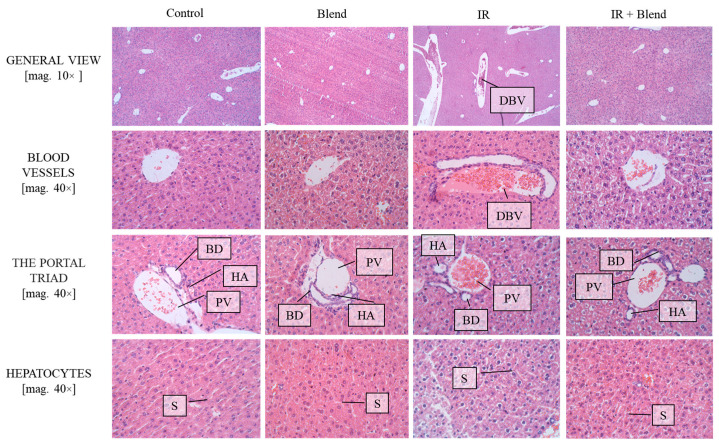
Microscopic sections of mouse liver tissues stained using the H&E method. Cell nuclei are visible as blue-purple areas, while the cytoplasm and extracellular structures appear pink–red. DBV—Dilated Blood Vessels; BD—Bile Ducts; HA—Hepatic Artery; PV—Portal Vein; S—Sinusoids. For each mouse, at least 10 images were taken from different microscopic fields at two magnifications fold (10× and 40×).

To observe tissue-level changes, liver and kidney samples were stained with H&E. Histological analysis of the liver revealed group-specific morphological differences, particularly in the portal triad and surrounding hepatocytes. Normal hepatic structure was observed in the control group, with intact portal triads, well-organized hepatocyte cords, and regular, unobstructed sinusoids (S), showing no signs of cellular stress or vascular congestion. In contrast, the IR group showed marked structural disruptions, including dilated blood vessels (DBV), vascular congestion, distorted portal triads, and hepatocellular stress with occasional cytoplasmic vacuolization and irregular hepatocyte arrangement, indicative of radiation-induced injury. The extract-supplemented irradiated group demonstrated partial preservation of liver morphology. Although mild sinusoidal dilatation and slight portal irregularities were noted, the portal triad components remained relatively intact, and sinusoidal structure was less affected with hepatocyte cords appearing more regularly organized than in the IR group. These observations suggest a protective effect of the blend against radiation-induced liver injury, with attenuation of vascular and cellular alterations and partial maintenance of normal tissue architecture (Figure 7).

**Figure 8:**
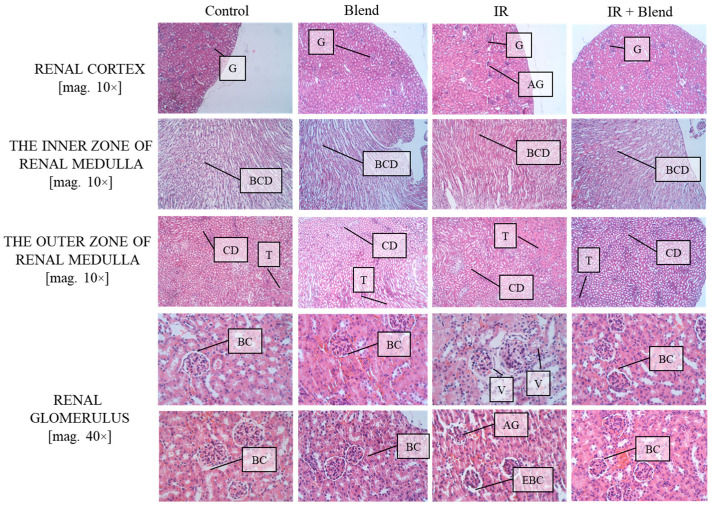
Microscopic sections of mouse kidney tissues stained using the H&E method. Cell nuclei are visible as blue-purple areas, while the cytoplasm and extracellular structures appear pink–red. G—Glomeruli; AG—Atrophic Glomeruli; BCD—Bellini Collecting Ducts; CD—Collecting Ducts; T—Tubule; BC—Bowman Capsule; V—Vacuolization; EBC—Enlarged Bowman Capsule. For each mouse, at least 10 images were taken from different microscopic fields at two magnifications fold (10× and 40×).

Histological evaluation of kidney sections revealed marked differences in tissue morphology across the experimental groups. In the control group, normal renal histology was observed, with well-preserved Bowman’s capsules (BC) and intact glomerular and tubular structures, showing no evidence of cellular stress, inflammation, or structural alterations. The IR group exhibited significant structural alterations, including expansion of Bowman’s capsules (EBC), vascular congestion (V), and apparent tubular degeneration, with widespread tubular vacuolization and irregular glomerular arrangement. Additionally, observed apoptotic glomerular cells (AG) and interstitial abnormalities indicated radiation-induced renal injury. In the irradiated group supplemented with the extract blend, renal tissue demonstrated notable attenuation of these damages. Although mild swelling and early degenerative changes were still present, the overall morphology of the kidney tissue, including Bowman’s capsules and glomerular structure, was comparatively well preserved relative to the IR group with fewer apoptotic cells and less vascular congestion (Figure 8). These observations support a protective effect of the blend against radiation-induced kidney injury, partially maintaining normal tissue architecture, as well as reducing cellular and vascular alterations.

## 3. Discussion

IR exerts cytotoxic effects not only on malignant tissues but also on surrounding healthy cells, frequently resulting in adverse side effects in cancer patients. Importantly, tissue damage is not limited to high-dose exposure. A growing body of evidence links IR-induced genomic instability and mutagenesis to the development of stochastic effects, which may also impact individuals exposed to low-dose radiation outside of therapeutic contexts [28]. Both chronic low-dose and acute high-dose IR exposures can cause progressive structural and functional deterioration in metabolically active organs. These effects are mediated by sustained oxidative stress, persistent DNA damage, immune dysregulation, chronic inflammation, and prolonged activation of profibrotic signaling pathways, including the transforming growth factor-beta (TGF-β) cascade [8,29].

Given the complex and multifactorial pathophysiology of radiation-induced tissue injury, increasing attention is being directed toward the development of adjunctive therapeutic agents capable of mitigating these harmful effects while maintaining a well-established safety profile. Among the most promising candidates are naturally occurring polyphenols, which are widely distributed in plants and known for their broad spectrum of bioactivities. Extensive preclinical evidence supports their antioxidant, anti-inflammatory, and anti-apoptotic properties [30]. Polyphenols exert their protective effects via multiple mechanisms, including the scavenging of ROS, modulation of redox-sensitive signaling pathways, and regulation of key components of both innate and adaptive immune responses. These pleiotropic actions underscore their potential as radioprotective agents, particularly in scenarios involving prolonged or repeated exposure to IR [31].

In the present study, we evaluated the radioprotective efficacy of a polyphenol-rich blend composed of chokeberry (*Aronia melanocarpa* L.), elderberry (*Sambucus nigra* L.), blackcurrant (*Ribes nigrum* L.), and evening primrose (*Oenothera biennis* L.) extracts in a murine model of acute IR exposure. Phytochemical profiling of the tested extract blend revealed a high content of polyphenolic compounds, including phenolic acids and flavonoids, with anthocyanins representing a prominent subclass. These compounds are widely recognized for their redox-modulating and immunomodulatory activities, impacting a variety of biological processes including apoptosis, platelet aggregation, vascular function, and xenobiotic metabolism [32].

Exposure of mice to 6 Gy of IR resulted in a significant reduction in peripheral blood leukocyte, lymphocyte, and platelet counts. Additionally, flow cytometric analysis revealed a decreased frequency of CD45^+^ cells in the bone marrow. It is well established that high-dose radiation can severely deplete hematopoietic stem and progenitor cells in the bone marrow as well as lymphocyte populations in lymphoid organs [33]. Disruption of hematopoietic and immune compartments contributes to immunosuppression, thereby increasing the risk of opportunistic infections, systemic malaise, and, in severe cases, radiation-induced mortality [34]. Accordingly, protecting the hematopoietic and lymphoid systems is essential to mitigate IR-induced lethality. In our study, pre-treatment with the polyphenol-rich extract blend attenuated radiation-induced reductions in peripheral blood and bone marrow CD45^+^ cells, indicating partial restoration of hematopoietic function. This observation is consistent with previous reports on the immunoprotective potential of polyphenolic compounds [35,36]. Fan et al. demonstrated that a 14-day pre-treatment with anthocyanins prior to 6 Gy gamma irradiation significantly reduced micronucleus formation in bone marrow polychromatic erythrocytes (PCEs), indicating immunostimulatory properties capable of mitigating radiation-induced immunosuppression [32]. Interestingly, despite the overall immunosuppressive effects of irradiation, an increase in splenic CD45^+^CD3^+^ T lymphocytes was observed in our study, with a further elevation in animals receiving the extract blend. Notably, this T-cell expansion was accompanied by a significant reduction in IL-1β expression in the spleen, particularly in the polyphenol-treated group. Given that radiation exposure induces inflammasome activation and IL-1β release in immune cells, including splenic T lymphocytes, the suppression of IL-1β expression in the spleen of polyphenol-treated mice may suggest effective inhibition of inflammation-related signaling pathways [37]. Collectively, these findings suggest that the blend exerts a dual immunomodulatory effect by promoting T-cell survival or expansion while simultaneously suppressing pro-inflammatory signaling in lymphoid tissues. The radiation-induced increase in splenic CD45^+^CD3^+^ T lymphocytes may reflect a systemic immune response to tissue injury in other organs [38], such as the bone marrow, where elevated TNF-α levels were detected. As a central lymphoid organ, the spleen may respond to these systemic inflammatory signals by mobilizing or retaining specific T-cell subsets [39].

Given their essential physiological functions, the liver and kidneys were selected as representative target organs for assessing systemic effects of IR. Although not classified as highly radiosensitive, both are susceptible to delayed radiation-induced injury, in which oxidative stress, inflammation, and cellular death play key pathogenic roles. These mechanisms are associated with the development of radiation-induced liver damage (RILD) and radiation nephropathy (RN), for which effective protective strategies remain limited [40,41].

In our study, gene expression analysis revealed distinct oxidative stress responses in the liver and kidneys of irradiated mice. In the liver, activation of antioxidant defenses against IR-induced stress was evidenced by upregulated expression of *Nrf2* and its downstream target *Nqo1*. As a central component of the Nrf2-mediated redox regulatory pathway, Nqo1 contributes to cellular protection by facilitating quinone detoxification and limiting ROS accumulation [42]. In the kidneys, increased expression of both pro-oxidant enzymes and the enzymatic antioxidant defense system was observed, indicating a coordinated response aimed at restoring redox balance. These findings are consistent with previous reports showing that IR leads to excessive production of ROS, and oxidative stress, which in turn induces simultaneous activation of pro-oxidant and antioxidant gene networks to mitigate redox imbalance [43,44]. Observed by us modulation of oxidative stress-related gene expression following treatment with the polyphenol-rich extract blend suggests a regulatory influence on molecular pathways involved in redox homeostasis, inflammation, and cell death. The blend’s ability to normalize the expression of both pro-oxidant and antioxidant genes may reflect its role in fine-tuning the cellular response to IR, preventing excessive ROS accumulation while supporting adaptive antioxidant mechanisms. Furthermore, elevated lipid peroxidation levels, a well-established biomarker of radiation-induced oxidative injury, in both organs confirmed the presence of oxidative damage [45]. The supplementation with the extract blend reduced the intensity of lipid peroxidation caused by ionizing radiation in liver and renal tissue, which may suggest that the extract blend effectively protects cellular membranes from radiation-induced oxidative damage.

Induction of oxidative stress by ionizing radiation can initiate inflammatory signaling pathways and cell death mechanisms. Excessive levels of ROS activate transcription factors such as NF-κB, promoting the expression of pro-inflammatory cytokines and adhesion molecules, and thereby sustaining a chronic inflammatory state [46]. Furthermore, persistent oxidative stress and inflammation can lead to the activation of programmed cell death pathways, including apoptosis and necrosis. Radiation-induced cell death contributes to tissue dysfunction and impaired regenerative capacity, thereby complicating recovery process [47]. The observed here downregulation of pro-inflammatory and pro-apoptotic gene expression in treated animals suggests that the polyphenol-rich blend may attenuate radiation-induced inflammatory signaling and limit activation of programmed cell death pathways. This multifaceted modulation implies a protective mechanism by which the tested formulation mitigates oxidative injury, suppresses chronic inflammation, and preserves tissue integrity following IR exposure. Numerous studies have confirmed that polyphenol-rich extracts exert protective effects against oxidative stress, inflammation, and cell death, supporting their potential as effective radioprotective agents [12,48,49,50,51].

Interestingly, the expression of *Sirt1*, a negative regulator of inflammatory responses and cellular senescence, was decreased following IR but restored by supplementation with the extract blend in the liver. This finding supports a potential mechanism through which the polyphenol-rich blend exerts anti-inflammatory and cytoprotective effects, by enhancing the SIRT1 axis, a finding consistent with literature reports highlighting the interplay between polyphenols, SIRT1, and cellular stress regulation. Zhang et al. demonstrated that resveratrol (3,5,4′-trihydroxy-trans-stilbene) protects hematopoietic stem cells (HSCs) from radiation at least in part, via activation of SIRT1 [48].

Histological analysis demonstrated preservation of hepatic and renal structure in irradiated mice treated with the polyphenol-rich blend, providing further evidence of its protective effects. Irradiated tissues from blend-supplemented mice displayed only minor abnormalities, whereas the IR group showed significant structural damage. These results imply that the blend confers noticeable tissue-level protection in addition to modifying molecular responses. Similarly, a study investigating quercetin treatment reported that irradiated rats exhibited liver and kidney histopathological damage, which was significantly ameliorated by quercetin supplementation, confirming its protective effects at the tissue level [52].

The results of this study demonstrate that the tested polyphenol-rich blend exerts significant radioprotective effects by attenuating oxidative damage, modulating inflammation and immune responses, as well as preventing cell death in in vivo condition. These findings underscore the potential of polyphenolic compounds as effective adjuvants in radiotherapy and as protective agents for individuals at risk of radiation exposure. However, it is important to consider potential long-term effects and limitations of the current study, such as the use of a single radiation dose and the short duration of observation. Based on these promising preclinical results, a clinical trial has been planned as the next phase of research to confirm the efficacy and safety of the blend in humans over longer follow-up periods.

## 4. Materials and Methods

### 4.1. Investigational Blend

The blend was prepared by Greenvit Ltd. (Zambrów, Poland), a supplier of plant extracts, by mixing pure extracts of chokeberry (*Aronia melanocarpa* L.), elderberry (*Sambucus nigra* L.), blackcurrant (*Ribes nigrum* L.) fruits, and evening primrose (*Oenothera biennis* L.) seeds in an appropriate percentage ratio. The blend was standardized to contain a minimum of 35% polyphenols, as determined by ultraviolet (UV) spectroscopy. The composition and ratio of the components were optimized using the Design of Experiments (DoE) software, (Design-Expert^®^ Version 12, Stat-Ease^®^, Minneapolis, MN, USA). The plant materials used for extract production were sourced from cultivated fields in Poland and other European countries. Extraction was performed using ethanol or water under mild conditions to preserve thermolabile compounds. The resulting liquid extracts were subsequently concentrated under reduced pressure and spray-dried. The blend is currently undergoing patent approval procedures.

#### Identification and Quantification of Anthocyanins and Phenolic Acids in Individual Extract and Their Blend

To characterize the phenolic profile of each individual extract and their blend, anthocyanins and total polyphenol content were analyzed using UPLC and spectrophotometric methods. Anthocyanin content was assessed using an ACQUITY UPLC I-Class PLUS System (Waters, Milford, MA, USA) equipped with a photodiode array (PDA) detector. Chromatographic separation was carried out on an ACQUITY UPLC^®^ BEH C18 column (2.1 mm × 150 mm, 1.7 μm; (Waters, Milford, MA, USA) maintained at 35 °C without a pre-column. The autosampler temperature was set to 5 °C. Extract and blend samples were dissolved in 0.01% formic acid in methanol/water (8:2, *v*/*v*) prior to analysis. The mobile phase consisted of 2% formic acid in water (component A) and acetonitrile (component B), with a flow rate of 0.3 mL/min. The gradient elution profile (relative to component A) was programmed as follows: 0–3.0 min, 96% (isocratic); 3.0–8.0 min, 96% to 85%; 8.0–11.0 min, 85% to 20%; 11.0–11.5 min, return to 96%. The equilibration time was 1.5 min. Anthocyanins were detected at 520 nm, and data were subsequently acquired and processed via the Empower Chromatography Data System (Empower 3 Software, Build Number: 3471, Waters, Milford, MA, USA).

Quantitative determination of anthocyanins was also conducted using UV-Vis spectrophotometry. Samples were diluted to 0.1 mg/mL in methanol/water (4:6, *v*/*v*), then further diluted in buffers at pH 1.0 and pH 4.5. After incubation for 5 min in the dark at room temperature, absorbance was measured at 520 and 700 nm using a NanoPhotometer^®^ NP80 (Implen GmbH, Munich, Germany). The concentration of anthocyanins was calculated based on the molecular weight and molar absorptivity of cyanidin-3-glucoside.

The total polyphenol content (TPC) in each individual extract, as reported by the supplier (Greenvit Ltd., Zambrów, Poland), was determined using UV spectrophotometry. TPC in the blend was determined using the Folin–Ciocalteu colorimetric method with caffeic acid (Sigma-Aldrich, St. Louis, MO, USA) as the standard. Blend samples were diluted in methanol/water (4:6, *v*/*v*) to a final concentration of 1 mg/mL. For each measurement, 1 mL of sample was mixed with 4 mL of water and 0.5 mL of Folin–Ciocalteu reagent. After 1 min, 2 mL of 20% (*w*/*v*) sodium carbonate solution (Sigma-Aldrich, Saint Louis, MO, USA) was added, and the mixture was diluted to 10 mL with water. Samples were incubated at room temperature for 30 min in the dark. Absorbance was measured at 760 nm using a NanoPhotometer^®^ NP80. Final concentrations were calculated based on a six-point calibration curve.

### 4.2. In Vivo Mouse Model

Female Balb/c mice, aged 10–11 weeks, were used in this study. The animals were acclimatized in a pathogen-free animal facility under standard laboratory conditions for one week prior to the experiments and were provided with water and standard chow ad libitum. The study protocol was approved by the Local Ethics Committee in Katowice (Silesian Medical University; approval no. 53/2022).

### 4.3. Experimental Design

The animals were randomly divided into four groups (10 animals per group): (I) unexposed control; (II) mice supplemented with the extract blend (100 mg/kg body weight); (III) mice exposed to irradiation; and (IV) mice supplemented with the extract blend and exposed to irradiation. Animals in groups II and IV were intragastrically supplemented with the extract blend at a dose of 100 mg/kg body weight, prepared in saline solution, while animals in groups I and III received intragastric saline solution. Supplementation was performed once daily for 7 days, with a maximum volume of 100 µL adjusted according to body weight. After this period, animals in groups III and IV were exposed to a single sub-lethal 6 Gy dose of IR using a Varian TrueBeam accelerator (Varian Medical Systems, Palo Alto, CA, USA). Prior to irradiation, the mice were anesthetized with a ketamine and xylazine mixture at a 19:1 ratio. At 24 h after irradiation, blood was collected from the cheek for analysis using a hematology analyzer (SCIL VET ABC PLUS+, Horiba, Warsaw, Poland). Supplementation with the blend was continued for 4 days post-irradiation in groups III and IV. All animals were sacrificed 4 days after irradiation. The mice were anesthetized by intraperitoneal administration of Avertin (0.5 mg/kg), followed by spinal cord dislocation. Tissues were collected for further analysis, including the liver, kidneys, bone marrow, and spleen. Bone marrow and spleen were used for cell isolation. The liver and kidneys were divided: one part was snap-frozen in liquid nitrogen and stored at −80 °C for further analysis, while the rest were fixed in formalin and embedded in paraffin for histological examination.

### 4.4. Cell Isolation from Spleen and Bone Marrow

Spleen (SPL) and bone marrow (BM) were collected for cell isolation. The spleen was immediately placed in a 60 mm Petri dish containing phosphate-buffered saline (PBS, Corning Life Sciences, Corning, NY, USA) supplemented with 10% fetal bovine serum (FBS, Corning Life Sciences, Corning, NY, USA) and 1% Penicillin–Streptomycin (Sigma-Aldrich, Saint Louis, MO, USA). The tissue was gently crushed using sterile glass slides, and the resulting suspension was collected using sterile Pasteur pipettes and transferred to conical tubes. The bone marrow was collected from the femurs and tibias using sterile Pasteur pipettes and transferred to conical tubes. The obtained suspensions from both the SPL and BM were filtered through a 70 μm nylon mesh filter (pluriSelect Life Science UG, Leipzig, Germany) and centrifuged at 300× *g* for 10 min at 4 °C. The supernatants were discarded. For spleen tissue, red blood cell (RBC) lysis was performed using distilled water. The RBC lysis and centrifugation steps were repeated until a clear pellet, free of RBCs, and a clear supernatant were obtained. The final cell pellets were resuspended in complete culture medium RPMI 1640 (Sigma-Aldrich, Saint Louis, MO, USA) supplemented with glutamine (Sigma-Aldrich, Saint Louis, MO, USA), 10% FBS, 0.1 mM Non-Essential Amino Acids (NEAA, (Sigma-Aldrich, Saint Louis, MO, USA)), 10 mM HEPES (Sigma-Aldrich, Saint Louis, MO, USA), 1 mM sodium pyruvate (Sigma-Aldrich, Saint Louis, MO, USA), and 1% Penicillin/Streptomycin. The cells count and viability were assessed using Trypan Blue (TB) (BioRad, Hercules, CA, USA) exclusion assay.

### 4.5. Flow Cytometry Analysis

Cells isolated from the spleen and bone marrow were cultured for 24 h, then stained with antibodies (APC/Cyanine7 anti-mouse CD45.2 Antibody, #109824; BioLegend^®^ FITC anti-mouse CD3 Antibody, #100204) at a dilution of 1:10 per 1 million cells for 30 min at 4 °C. After staining, cells were washed, centrifuged at 200× *g*, and fixed in 1% paraformaldehyde. The analysis of the samples was performed using a BD FACSCanto^TM^ II cytometer (BD Biosciences, San Jose, CA, USA).

### 4.6. TNF-α Measurement in Bone Marrow

Isolated bone marrow cells were seeded into 24-well plate at a density of 3 million cells per well and cultured for 24 h. After this stabilization period, the cells were stimulated with phorbol myristate acetate (PMA, Sigma-Aldrich, Saint Louis, MO, USA) and ionomycin (Sigma-Aldrich, Saint Louis, MO, USA) at concentrations of 50 ng/mL and 1 µg/mL, respectively, for 5 h. Following incubation, supernatants were collected to measure TNF-α levels using the ELISA test (Mouse TNF-alpha DuoSet ELISA, #DY410, R&D Systems—Biotechne^®^, Minneapolis, MN, USA).

### 4.7. RNA Isolation and Gene Expression Analysis

The liver and kidney tissues were sectioned into 50 mg samples. RNA was extracted using phenol/chloroform according to the protocol provided with the Total RNA Mini Kit (A&A Biotechnology^©^, Gdańsk, Poland). RNA sample concentration and purity were assessed using a NanoPhotometer^®^ NP80 spectrophotometer. Purity was determined using the 260/280 nm and 260/230 nm absorbance ratios, with acceptable ranges of ~2.0 and 2.0–2.2, respectively.

The isolated RNA was reverse-transcribed into cDNA using the RT-PCR method (High-Capacity cDNA Reverse Transcription Kit; Applied Biosystems^TM^, Foster City, CA, USA). qRT-PCR was performed using the PerfeCTa SYBR^®^ Green FastMix Reagent (Quantabio©, Beverly, MA, USA) on the Rotor-Gene Q real-time cycler (Qiagen, Hilden, Germany). The PCR protocol consisted of an initial denaturation step at 95 °C for 30 s; followed by 40 cycles of 95 °C for 5 s, 60 °C for 30 s. The specificity of the PCR products and the detection of potential primer–dimer formations or non-specific amplifications were confirmed using melting curve analysis and agarose gel electrophoresis. The relative quantitative determination of gene expression was performed using the 2^−ΔΔCt^ method, with Tbp (TATA-binding protein) gene as the reference for the liver and kidneys and *Actb* (β-actin) as the reference gene for the spleen. The sequences of the primers used in the qPCR reaction are presented in Table 3.

### 4.8. Lipid Peroxidation

Lipid peroxidation was evaluated using the commercially available Lipid Peroxidation (MDA) Assay Kit (Merck KGaA, Darmstadt, Germany, #MAK568). Approximately 50 mg of mouse liver and kidney tissues were homogenized in MDA Lysis Buffer. The MDA concentration in the resulting tissue supernatants was determined by conducting a reaction with thiobarbituric acid (TBA) at 95 °C, resulting in the formation of a colored MDA-TBA_2_ adduct. Absorbance was measured at 532 nm using the EnVision 2103 Multilabel Reader (PerkinElmer, Waltham, MA, USA).

### 4.9. Histological Sample Preparation and Evaluation

Tissue sections from the kidneys and liver were subjected to hematoxylin and eosin (H&E) staining using the H&E Staining Kit (Abcam, Cambridge, UK, #ab245880). Slides were deparaffinized and rehydrated by sequential immersion in Xylene I (8 min), Xylene II (7 min), which are two consecutive immersions in fresh portions of the same xylene to ensure complete removal of paraffin and proper tissue permeability, followed by graded ethanol solutions (100%, 80%, and 70%; 5 min each), followed by washing in running tap water. Hematoxylin (Mayer’s) was applied for 5 min, then rinsed with distilled water. A bluing reagent was added for 10–15 s, followed by another rinse. Slides were dipped in absolute alcohol and blotted, stained with Eosin Y for 2–3 min, and rinsed again with absolute alcohol. Dehydration was completed with three changes of absolute alcohol, cleared with Xylene (two dips each), and mounted with synthetic resin. A decolorization step was performed on two sections from each mouse. For each preparation, at least 15 images were captured from different microscopic fields at two magnification levels (10× and 40×).

### 4.10. Statistical Analysis

Data were analyzed using GraphPad Prism10.2.3 software (GraphPad Software, San Diego, CA, USA). The normality of the data distribution was assessed using the Shapiro–Wilk test, with a *p*-value < 0.05 considered evidence of a significant deviation from normality. When distributions were normal (*p* ≥ 0.05), comparisons were performed using Student’s *t*-test; otherwise, the non-parametric Mann–Whitney U test was applied. Results are presented as mean ± standard deviation (SD) for parametric tests and as median (Q1–Q3) for non-parametric tests, Statistical significance was set at *p* < 0.05.

## 5. Conclusions

This study demonstrates that the polyphenol-rich extract blend composed of chokeberry, elderberry, blackcurrant, and evening primrose significantly mitigates the harmful effects of acute IR in a murine model. The blend effectively preserved hematological parameters, supported immune cell viability, reduced the expression of pro-inflammatory cytokines, and modulated gene expression related to oxidative stress and apoptosis. Histological analyses further confirmed its protective effects at the tissue level. These findings highlight the potential of the tested formulation as a natural radioprotective agent and support its future development as an adjuvant strategy for individuals undergoing radiotherapy or those occupationally exposed to IR.

## Figures and Tables

**Table 1:** Main anthocyanins identified in the blend and their contents.

Compound	Retention Time [min]	Main Botanical Source in Blend	Relative% of Anthocyanins (est.)	Estimated Content in Blend (% *m*/*m*, Based on 20.4%)
C-3,5-di-Glu	7.561	Elderberry	4.35%	0.89%
C-3-Sam-5-Glu		Elderberry		
D-3-Glu	8.017	Blackcurrant	4.40%	0.90%
D-3-Rut	8.245	Blackcurrant	5.11%	1.04%
C-3-Gal	8.509	Chokeberry	17.41%	3.55%
C-3-Sam	8.733	Elderberry	34.33%	7.01%
C-3-Glu	8.852	Elderberry + Blackcurrant	24.77%	5.05%
C-3-Rut	9.045	Blackcurrant	3.15%	0.64%
C-3-Ara	9.225	Chokeberry	5.45%	1.11%
C-3-Xyl	10.044	Chokeberry	1.03%	0.21%
Total			100.00%	20.40%

**Table 2:** The total phenolic content in individual extracts and their blend.

Compound	Total Phenolic Content% (*m*/*m*)
Chokeberry	70.86%
Elderberry	46.58%
Blackcurrant	47.11%
Evening primrose	50.99%
The blend	54.74%

**Table 3:** The sequences of primers used in the qPCR.

Gene	Primer Forward	Primer Reverse
*Tbp*	CAAACCCAGAATTGTTCTCCTT	ATGTGGTCTTCCTGAATCCCT
*Actb*	CACCCGCGAGCACAGCTTCTTT	TTGTCGACGACCAGCGCAGCGATA
*Nrf2*	CGAGATATACGCAGGAGAGGTAAGA	GCTCGACAATGTTCTCCAGCT
*Nqo1*	TTCTCTGGCCGATTCAGAG	GGCTGCTTGGAGCAAAATAG
*Nox4*	TGTTGGGCCTAGGATTGTGTT	AGGGACCTTCTGTGATCCTCG
*Ho-1*	CGTGCTCGAATGAACACTCT	GGAAGCTGAGAGTGAGGACC
*Gpx1*	GACACCAGGAGAATGGCAAGA	ACCATTCACTTCGCACTTCTCA
*Cat*	AGCGACCAGATGAAGCAGTG	TCCGCTCTCTGTCAAAGTGTG
*Sod1*	GGTGAACCAGTTGTGTTGTCAGG	ATGAGGTCCTGCACTGGTACAG
*Sod2*	TAACGCGCAGATCATGCAGCTG	AGGCTGAAGAGCGACCTGAGTT
*Sirt1*	GGAGCAGATTAGTAAGCGGCTTG	GTTACTGCCACAGGAACTAGAGG
*Nf-κB p65*	TGACCCCTGTCCTCTCACATCCG	CAGCTCCCAGAGTTCCGGTT
*Il-1β*	TCGTGCTGTCGGACCCATAT	GGTTCTCCTTGTACAAAGCTCATG
*Il-6*	CTACCCCAATTTCCAATGCT	ACCACAGTGAGGAATGTCCA
*Casp1*	GGCACATTTCCAGGACTGACTG	GCAAGACGTGTACGAGTGGTTG
*Hmgb-1*	GGGTCACATGGATTATTAGTGTGT	CAGGGCATGTGGACAAAA
*Icam-1*	CCATCACCGTGTATTCGTTTCC	CTGGCGGCTCAGTATCTCCTC
*Ifnγ*	CTGGCAGGATGATTCTGCTGG	GCATACGACAGGGTTCAAGTTAT
*Casp3*	GGAGTCTGACTGGAAAGCCGAA	CTTCTGGCAAGCCATCTCCTCA
*Bax*	TGCAGAGGATGATTGCTGAC	GATCAGCTCGGGCACTTTAG
*Bcl-2*	CTGGCATCTTCTCCTTCCAG	GACGGTAGCGACGAGAGAAG

## Data Availability

All data supporting the findings in this study will be made available on request.
